# Supplementary material for: Cardiac Remodeling in the Absence of Cardiac Contractile Dysfunction Is Sufficient to Promote Cancer Progression
Source: Cells. 2022 Mar 25;11(7):1108. doi: 10.3390/cells11071108 (PMC8997578; doi:10.3390/cells11071108)
Supplement: Supplementary file 1 [file cells-11-01108-s001.zip › cells-1634714-supplementary.pdf]

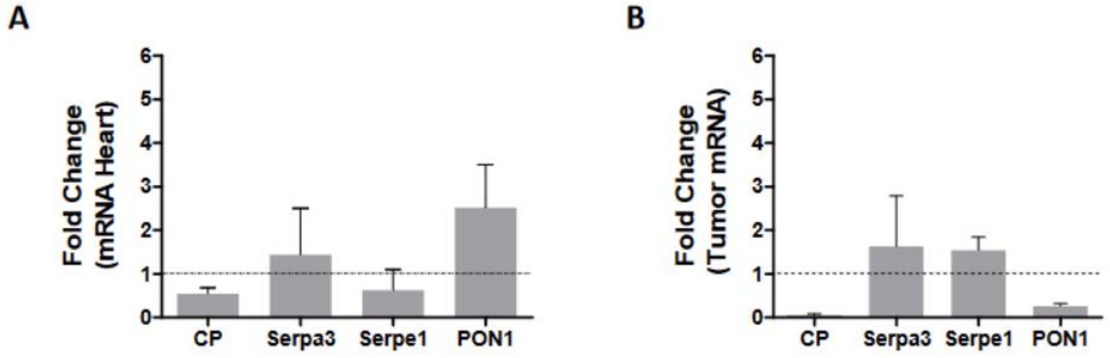

.

Supplementary Figure S1. Putative secreted factors that were found to be expressed alike in the PE-infused model. A B mRNA levels from either heart tissue (**A**) or tumor (**B**) of the indicated genes were measured by qRT-PCR. Data are presented as relative expression compared with control determined as 1. Data are presented as mean ±SEM and analyzed by multiple Student t tests. * $p < 0.05$. ** $p <0.01$. *** $p <0.001$.

Supplementary Table S1. Oligonucleotides used for qRT-PCR.

Gene	Forward	Reversed
HSP90	TCGTCAGAGCTGATGATGAAGT	GCGTTTAACCCATCCAACTGAAT
GAPDH	TTGCCATCAACGACCCCTTCAT	AGACTCCACGACATACTCAGCA
ANP	GCTTCCAGGCCATATTGGAG	GGGGGCATGACCTCATCTT
BNP	GAGGTCACTCCTATCCTCTGG	GCCATTTCCTCCGACTTTTCTC
ACTA1	GTGAGATTGTGCGCGACATC	GGCAACGGAAACGCTCATT
Col1a	CTGGCGGTTCAGGTCCAAT	TTCCAGGCAATCCACGAGC
TGFb3	CCTGGCCCTGCTGAACTTG	GACGTGGGTCATCACCGAT
ACTA2	GTCCCAGACATCAGGGAGTAA	TCGGATACTTCAGCGTCAGGA
FN	ATGTGGACCCCTCCTGATAGT	GCCCAGTGATTTCAGCAAAGG
POSTN	CCTGCCCTTATATGCTCTGCT	AAACATGGTCAATAGGCATCACT
CTGF	AGACCTGTGGGATGGGCAT	GCTTGGCGATTTTAGGTGTCC
PON1	GCTGAAGACTTAGAGATTCTGCC	GTTTACCACCAGTAGGTACACAG
Serpina3	AGAGGAGCTAAACCTGCCCAA	ATACGGCCTTACGAATGCCAC
Serpine1	CACCGTGAAGGTGCCTATGATG	GGCATTGCCCAGGTATTTCATC
CP	CTTAGCCTTGGCAAGAGATAAGC	GGCCTAAAAACCCTAGCCAGG

Supplementary Table S2. Echocardiography parameters for WT C57Bl6 mice: baseline (WT), tumor bearing (WT + Cancer) and PE infused tumor bearing (PE pumps + Cancer)

	**WT**	**WT+Cancer**	**PE pumps + Cancer**
IVSd;d-D	0.82 ± 0.004	0.83 ± 0.09	0.83 ± 0.12
LVID;d-D	3.6 ± 0.27	3.81 ± 0.14	3.47 ± 0.24
LVID;s-D	2.39 ± 0.04	2.48 ± 0.04	2.25 ± 0.18
LVPW;d-D	0.77 ± 0.07	0.79 ± 0.06	0.77 ± 0.08
FS (%)	33.35 ± 3.82	34.82 ± 1.84	35.07 ± 2.68
